# Deskewed LiDAR Odometry for Quadruped Robots in Environments with Varying Elevation

**DOI:** 10.3390/s26113518

**Published:** 2026-06-02

**Authors:** Eunhui Han, Heoncheol Lee

**Affiliations:** Department of IT Convergence Engineering, Kumoh National Institute of Technology, Gumi 39177, Republic of Korea; silverhee@kumoh.ac.kr

**Keywords:** LiDAR odometry, quadruped robot, motion deskewing, iterative closest point, *z*-axis observability, point-cloud registration

## Abstract

As robotics technology advances, quadruped robots have become capable of operating in complex environments with varying elevation, including ramps and level changes that are challenging for conventional wheeled platforms. While this terrain adaptability opens new opportunities for inspection, rescue, and exploration tasks, the repetitive impacts, frequent ground-contact transitions, and abrupt postural changes inherent to legged locomotion pose significant challenges for LiDAR odometry. High-frequency gait vibrations and abrupt attitude changes introduce intra-scan motion distortion that conventional single-twist deskewing cannot adequately suppress. In addition, sparse vertical geometric constraints in elevation-varying environments weaken *Z*-axis observability, allowing vertical drift to corrupt the horizontal pose estimate through Hessian coupling. To address these failure modes within a LiDAR-only framework, we propose a Piecewise-Constant Velocity deskewing scheme that partitions each scan into multiple temporal segments with safety clamping on vertical and attitude components, together with a two-stage ICP that decouples SE(3) optimization into horizontal (*x*, *y*, *yaw*) and vertical (*z*, *roll*, *pitch*) stages and applies observability-aware weighting in the vertical update. The proposed odometry front-end is evaluated on four real-world sequences collected with a Unitree Go2 quadruped robot equipped with a Velodyne VLP-16 LiDAR. Experimental results show consistently lower Absolute Pose Error (APE) than ICP, KISS-ICP, and F-LOAM across all sequences. Vertical drift suppression is most pronounced in the ramp-containing sequences, where baseline methods exhibit substantial *Z*-axis divergence.

## 1. Introduction

LiDAR odometry is a core front-end component for autonomous mobile robots, providing incremental pose estimation from sequential 3D point clouds without relying on external positioning infrastructure. Owing its robustness to illumination changes and its ability to provide accurate three-dimensional range measurements, LiDAR-based odometry has been widely adopted in robot navigation. In practical robotic systems, the accuracy of front-end odometry directly governs drift accumulation [1] and strongly affects downstream modules, including mapping and long-term navigation. In particular, accumulated drift can degrade localization reliability in environments with repetitive structures [2].

Quadruped robots have attracted growing interest for inspection, rescue, and exploration tasks because they can traverse unstructured terrain such as stairs, ramps, and level changes that are difficult for wheeled platforms. However, physical traversability does not guarantee reliable pose estimation. During locomotion, quadrupeds experience repeated foot–ground impacts, gait-induced body oscillations, and abrupt attitude changes that severely undermine the spatiotemporal consistency of LiDAR measurements, violating the static-pose assumption commonly used during scan acquisition. These dynamics induce intra-scan motion distortion, degrade point correspondence quality, and destabilize front-end estimation, making registration failures and drift accumulation more likely than on wheeled platforms. Similar effects have also been reported on dynamically unstable platforms, where abrupt attitude changes cause unintended LiDAR observations and degrade scan matching stability [3].

These challenges are particularly critical for vertical registration and *Z*-axis stability. In environments with varying elevation, small front-end errors can propagate into *pitch*/*roll* bias and accumulated vertical inconsistency. This instability is further compounded by both sensor and environmental factors. Multi-channel LiDAR sensors with sparse vertical resolution inherently weaken *Z*-axis observability, while dominant structures in stair, ramp, and uneven-terrain environments often provide limited sensitivity to vertical displacement in point-to-plane registration. As a result, horizontal constraints dominate the optimization, whereas vertical components remain underconstrained, causing recurrent *Z*-axis drift.

Existing LiDAR odometry methods have been developed primarily for wheeled or handheld platforms. Feature-based methods such as LOAM [4] and F-LOAM [5] rely on edge and planar feature extraction, which can degrade under the high-frequency vibrations of legged locomotion. IMU-aided front-end methods such as FAST-LIO2 [6] enable direct intra-scan deskewing, but their performance depends on IMU quality and calibration. Moreover, the impulsive dynamics of quadruped locomotion can amplify IMU integration errors. LiDAR-only methods such as KISS-ICP [7] assume a constant-velocity motion model, which becomes unreliable under abrupt attitude changes. Furthermore, single-stage SE(3) optimization in these methods allows vertical instability to propagate into all pose components.

This paper proposes an improved LiDAR odometry method for quadruped robots operating in unstructured terrain with varying elevation. The main contributions are as follows:

- Quadruped-Aware Deskewing: We propose a Piecewise-Constant Velocity deskewing method tailored to quadruped high-frequency vibration and abrupt attitude changes, and introduce *Z*-axis motion clamping to suppress early-interval distortion.
- Decoupled Registration for Z-Axis Drift Mitigation: We design a two-stage ICP framework that decomposes SE(3) optimization into separate horizontal and vertical stages, preventing vertical instability from corrupting horizontal pose estimates.
- Observability-Based Damping in Vertical Optimization: We introduce point-to-plane constraints with observability-score-based damping in the vertical stage to enhance *Z*-axis pose estimation robustness in environments with elevation changes.

## 2. Related Work

LiDAR odometry estimates the 6-DoF pose of a sensor through registration between consecutively acquired 3D point clouds. The most widely adopted registration framework is the ICP (Iterative Closest Point) family, and recent studies differ in (i) residual formulation (point-to-point/point-to-plane), (ii) map representation (voxel/surfel/implicit surface), (iii) scan distortion correction (deskewing), and (iv) handling of degeneracy and observability. This work adopts a LiDAR-only design philosophy, targeting scan distortion and *z*-axis drift.

### 2.1. LiDAR Registration and Odometry

ICP constructs correspondences between two point clouds and iteratively estimates the rigid-body transformation that minimizes the defined objective function [8]. Convergence characteristics and noise sensitivity vary with the residual formulation and data association strategy [9]. LOAM [4] performs separate scan-to-scan odometry and scan-to-map registration using edge and planar features, and F-LOAM [5] retains this pipeline while reducing computational overhead. LeGO-LOAM [10] incorporates ground segmentation to improve efficiency on uneven terrain, though this segmentation step has been reported to under-segment in non-flat or cluttered scenes [11]. More broadly, the curvature thresholds used for feature discrimination in LOAM-family methods are sensitive to sensor resolution and scene geometry [12], which can reduce robustness under unstable sensing and geometric conditions. In the dense representation category, SuMa performs frame-to-model ICP using a surfel-based map with projective data association [13], IMLS-SLAM reduces drift via scan-to-model registration on an implicit IMLS surface [14], and MULLS proposes a unified LiDAR odometry framework based on multi-metric linearized ICP, while further incorporating back-end pose graph optimization (PGO) [15]. These methods, however, depend on the quality of normal/surface estimation [13,14], and projective association can be vulnerable to abrupt viewpoint changes [13], requiring additional consideration in environments with frequent *roll*/*pitch* variation such as legged robots. KISS-ICP presents a LiDAR-only pipeline combining point-to-point ICP with adaptive correspondence thresholding, a robust kernel, constant-velocity (CV) deskewing, and voxel downsampling, eliminating data-dependent preprocessing stages [7]. Nevertheless, in quadruped locomotion, the approximation error of a single CV model can grow due to high-frequency vibrations and abrupt pose changes, and in environments with varying elevation, *z*-axis observability may weaken, causing vertical estimation errors to accumulate coupled with other DoF updates.

### 2.2. Scan Motion Distortion Correction (Deskewing)

Rotating LiDARs acquire each point within a sweep at a different timestamp; sensor motion during a sweep therefore introduces intra-scan motion distortion that degrades registration quality [4]. KISS-ICP demonstrates that a CV model alone can provide practical deskewing [7], but model mismatch accumulates when velocity or attitude changes abruptly within a sweep. CT-ICP integrates a continuous-time trajectory into the registration process to improve robustness to high-frequency motion [16], at the cost of increased optimization variables and computational complexity. IMU-coupled approaches (LIO-SAM [17], FAST-LIO2 [6]) enable direct intra-scan deskewing under high-dynamic conditions but depend structurally on IMU quality, time synchronization, and extrinsic calibration. Retaining the LiDAR-only philosophy [7], this work introduces Piecewise-Constant Velocity (PCV) deskewing, which partitions the sweep into time segments and approximates motion as constant within each segment, and combines it with *z*-axis motion clamping to suppress instantaneous vertical displacement induced by the gait cycle, thereby blocking the propagation of locomotion-induced distortion to registration quality.

### 2.3. LiDAR Odometry for Legged/Quadruped Robots

Quadruped robots are prone to registration degeneracy and distortion accumulation due to large *roll*/*pitch* variations, repetitive foot impacts, and discontinuous ground contact, leading to increased deskewing errors, degraded correspondence quality, and weakened *z*-axis constraints [18,19,20]. Ramezani et al. proposed a LiDAR odometry system for legged robots combining inertial–kinematic state-estimator-based scan accumulation, ICP registration, and learning-based loop closure [18]. VILENS by Wisth et al. tightly couples vision–IMU–LiDAR–leg odometry in a factor graph to maintain robustness under individual sensor degeneracy, reporting mitigation of vertical drift accumulation through online velocity bias estimation [19]. Additional works including Leg-KILO [20], LIKO [21], LLIO [22], and Okawara et al. [23] propose tightly coupled LiDAR–IMU–kinematics/contact frameworks for robust estimation under high-dynamic gait conditions. While these approaches demonstrate that proprioceptive information can substantially reinforce observability constraints [20,21,22,23,24], they introduce increased sensor dependency and calibration burden [19,20], and constraint validity varies with contact state estimation and ground model quality [22,24]. This work targets the suppression of locomotion-induced scan distortion and *z*-axis instability directly in the front-end registration stage, while preserving a LiDAR-only structure without additional sensors.

Although LiDAR-inertial and LiDAR-inertial–kinematic methods provide strong robustness for legged robots, this work intentionally focuses on a LiDAR-only odometry front-end. The goal is not to replace tightly coupled multi-sensor systems, but to isolate and improve the robustness of LiDAR registration itself under quadruped locomotion. If IMU measurements were incorporated, the deskewing prior and attitude information would be externally provided by the inertial sensor, making it difficult to isolate whether the observed improvement comes from the proposed motion compensation and vertical stabilization strategies or from the additional inertial information. Moreover, IMU-aided methods introduce additional dependencies, including accurate time synchronization, LiDAR–IMU extrinsic calibration, and reliable bias/noise modeling. Therefore, this study compares against LiDAR-based baselines under the same input modality and evaluates how much the LiDAR-only front-end can be stabilized without relying on additional sensing modalities.

### 2.4. Degeneracy and Observability-Based Stabilization

Point-cloud registration becomes numerically degenerate when the local geometry fails to provide sufficient constraints along certain degrees of freedom (e.g., long corridors, repetitive structures). Nobili et al. proposed a framework for predicting alignment risk based on overlap and geometric constraint analysis [25]; Zhang et al. presented a remapping-based stabilization method that identifies degenerate directions via Hessian spectral analysis and restricts updates to well-constrained subspaces [26]; Zhen and Scherer proposed a localizability quantification model for geometrically deficient scenes [27]; Hinduja et al. extended direction-selective updates to graph optimization via partial factor construction [28]; Tuna et al. proposed X-ICP, which analyzes per-direction alignment strength from correspondences and enforces it as a constraint [29]; and Hatleskog and Alexis proposed a degeneracy-aware ICP that continuously attenuates per-direction updates based on Hessian-based degeneracy estimation [30]. On the mapping side, OR-LIM proposes an observability-aware design that evaluates per-frame observability and incorporates it into map updates [31]. Regarding robustification, Chebrolu et al. proposed an adaptive robust kernel that automatically adjusts to the residual distribution [32]. KISS-ICP combines adaptive thresholding and a robust kernel to suppress outliers [7], but when the environment geometry structurally fails to constrain certain DoFs, erroneous updates along weakly constrained directions can still occur [26,28,29]. This work focuses on the intermittent weakening of *z*-axis observability in environments with varying elevation encountered by legged robots. A two-stage ICP structure structurally blocks the propagation of vertical instability to horizontal pose estimation, and in the decoupled vertical stage, point-normal-geometry-based observability scoring is directly applied as damping weights for vertical updates.

## 3. Proposed Method

**Figure 1:**
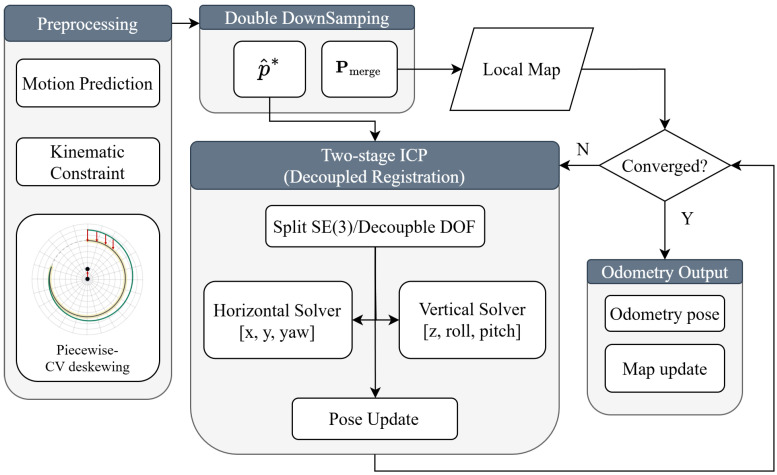
Overview of the proposed LiDAR odometry front-end pipeline.

Quadruped locomotion introduces two compounding challenges for LiDAR odometry: intra-scan motion distortion caused by high-frequency gait vibrations, and intermittent *Z*-axis observability degradation on slopes and stairs that destabilizes the full 6-DoF pose estimate. As illustrated in Figure 1, the proposed front-end addresses these challenges through two targeted modifications. In the motion compensation stage, a Piecewise-Constant Velocity deskewing scheme with *Z*-axis motion clamping suppresses locomotion-induced scan distortion before registration. In the registration stage, SE(3) optimization is restructured into a horizontal solver (*x*, *y*, *yaw*) and a vertical solver (*z*, *roll*, *pitch*) with observability-aware weighting, preventing vertical instability from contaminating horizontal pose estimation.

**Figure 2:**
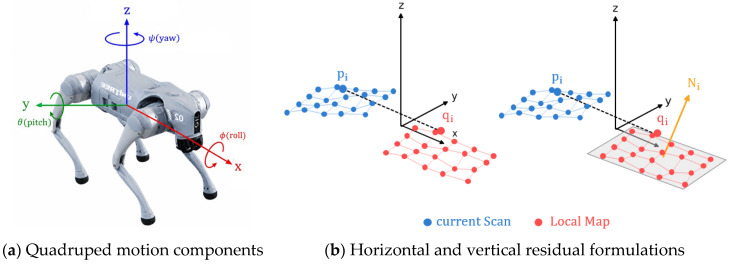
Conceptual illustration of quadruped motion components and two-stage ICP residuals.

Figure 2 illustrates the motion components and residual formulations used in the proposed two-stage registration. As shown in Figure 2a, quadruped motion is represented by translation along *x*, *y*, and *z* and rotation in *roll*, *pitch*, and *yaw*. Based on this representation, the proposed method separates the pose update into horizontal and vertical components. Figure 2b shows that the horizontal stage uses point-to-point residuals to estimate *x*, *y*, and *yaw*, whereas the vertical stage uses point-to-plane residuals with local normals estimated from neighboring map points to refine *z*, *roll*, and *pitch*. The following subsections formalize this decomposition and describe each component in detail.

### 3.1. Problem Formulation and State Decomposition

A single LiDAR scan is defined as

(1) $$P={\left\{(p_{i},\tau_{i})\right\}}_{i=1}^{N},$$

where $p_{i}\in R^{3}$ is the *i*-th point and $\tau_{i}\in [0,1]$ is the normalized acquisition time within the scan. The pose of the current frame is represented as T∈SE(3), and its incremental update is written as

(2) $$\delta \xi ={\left[\begin{array}{cccccc} \delta x & \delta y & \delta z & \delta \phi & \delta \theta & \delta \psi \end{array}\right]}^{⊤}.$$

Here, δξ is treated as a twist-like incremental pose update in SE(3), where $\left(\delta x,\delta y,\delta z\right)$ represent translational increments and $\left(\delta \phi ,\delta \theta ,\delta \psi \right)$ represent *roll*, *pitch*, and *yaw* increments, respectively. Throughout this paper, the variable ordering of the full state vector is fixed as ${\left[\delta x,\delta y,\delta z,\delta \phi ,\delta \theta ,\delta \psi \right]}^{⊤}$, and all subsequent Jacobian, Hessian, and state update expressions follow this ordering. In conventional coupled 6-DoF registration, this full state is estimated simultaneously in a single optimization problem. However, on slopes and level changes, the observability of *z*, *roll*, and *pitch* may weaken or change abruptly, so the instability of the vertical state can propagate to the horizontal state through the coupling terms of the Hessian.

To make this more explicit, the horizontal and vertical states are defined from the full state vector as

(3) $$\delta \xi_{H}={\left[\begin{array}{ccc} \delta x & \delta y & \delta \psi \end{array}\right]}^{⊤},\;\delta \xi_{V}={\left[\begin{array}{ccc} \delta z & \delta \phi & \delta \theta \end{array}\right]}^{⊤},$$

where $\delta \xi_{H}$ and $\delta \xi_{V}$ are partial state vectors that extract only the horizontal components $\left(x,y,\psi \right)$ and vertical components $\left(z,\phi ,\theta \right)$ from δξ, respectively. Viewing the state in horizontal–vertical order to clarify the block structure, the coupled normal equation can be expressed conceptually as

(4) $$\left[\begin{array}{cc} H_{HH} & H_{HV}\\ H_{VH} & H_{VV} \end{array}\right]\left[\begin{array}{c} \delta \xi_{H}\\ \delta \xi_{V} \end{array}\right]=-\left[\begin{array}{c} g_{H}\\ g_{V} \end{array}\right],$$

where $H_{HH}$ and $H_{VV}$ are the self-coupling terms for the horizontal and vertical states, respectively, and $H_{HV}$, $H_{VH}$ denote the coupling between the two states. When vertical observability decreases, $H_{VV}$ can become ill-conditioned, and as a result, the horizontal state is also destabilized through the coupling terms $H_{HV}$ and $H_{VH}$. That is, insufficient information in the vertical state leads not only to degraded estimation of *z*, *roll*, and *pitch*, but also to reduced stability of the full pose estimate including *x*, *y*, and *yaw*. It should be noted that this decomposition is not based on an assumption that the horizontal and vertical motions are physically independent or weakly coupled. On sloped terrain, horizontal translation, vertical displacement, and attitude changes are geometrically related. However, in LiDAR-only quadruped odometry, the vertical components can become intermittently weakly constrained due to sparse vertical LiDAR resolution, gait-induced motion, and elevation-varying geometry. Under such conditions, a joint SE(3) update may allow uncertainty in the vertical subspace to affect the horizontal update through the coupling blocks of the Hessian. Therefore, the proposed decomposition is introduced as a stabilization-oriented separation of update variables, aiming to regulate the propagation of poorly constrained vertical corrections into the horizontal pose estimate.

Rather than solving this coupled structure directly, the proposed method adopts a decoupled optimization structure in which the horizontal state $\delta \xi_{H}$ is estimated first in a stable manner, and the vertical state $\delta \xi_{V}$ is then corrected separately. This state decomposition serves as the direct design basis for the decoupled two-stage ICP proposed in Section 3.3, and is realized as a sequential estimation structure that first secures horizontal pose stability and then conditionally refines the vertical pose. This decomposition corresponds to the horizontal and vertical registration branches of the two-stage ICP module shown in Figure 1.

### 3.2. Quadrupedal-Motion-Aware Piecewise-Constant Velocity Deskewing

A quadrupedal robot undergoes repeated foot contacts and liftoffs throughout its gait cycle, generating high-frequency vibrations and instantaneous *pitch*/*roll* changes in the body frame. These motions can induce substantial intra-scan distortion even within a single LiDAR scan acquisition window, making it difficult to accurately compensate for them under a single constant-twist assumption. To address this, the proposed method partitions a single scan into multiple short temporal segments. Let K denote the number of such segments. In our implementation, the Velodyne VLP-16 LiDAR was operated at approximately 10 Hz, and the odometry pose was updated once per incoming scan. We set K = 4 for PCV deskewing, corresponding to an approximate segment duration of 0.025 s per scan segment.

Let the predicted poses at the start and end of the scan be $T_{s},\;T_{f}\in SE(3)$, respectively. The relative motion between the two poses is defined as

(5) $$\Delta T=T_{s}^{-1}T_{f},\;\xi =log(\Delta T),$$

where ξ∈se(3) is the twist representation for the full scan interval. However, in quadrupedal environments, the raw motion prior may contain brief vertical spikes or *roll*/*pitch* transients; using it directly could amplify abnormal distortion during deskewing. Therefore, a corrected twist $\tilde{\xi }$ is constructed by clamping the vertical translation and attitude components:

(6) $$\tilde{\xi }={\left[\begin{array}{cccccc} \xi_{x} & \xi_{y} & clip(\xi_{z};-\zeta_{z},\zeta_{z}) & clip(\xi_{\phi };-\zeta_{r},\zeta_{r}) & clip(\xi_{\theta };-\zeta_{p},\zeta_{p}) & \xi_{\psi } \end{array}\right]}^{⊤},$$

where $\zeta_{z}$, $\zeta_{r}$, and $\zeta_{p}$ denote the allowable limits for vertical translation, *roll*, and *pitch*, respectively. Importantly, *x*, *y*, and *yaw* are retained because they reflect the actual locomotion direction during walking, whereas only z, *roll*, and *pitch*, which are more susceptible to gait-induced oscillations and abrupt attitude changes in quadruped locomotion, are restricted.

In this work, the clamping thresholds were set to $\zeta_{z}$ = 0.5 m, $\zeta_{r}$ = 0.2 rad, and $\zeta_{p}$ = 0.2 rad, and the same values were used across all evaluated sequences without sequence-specific tuning. These values were selected in a platform-aware manner by considering the LiDAR scan period, body-height oscillation, and attitude variation of the Unitree Go2 during walking and ramp traversal. However, they were intentionally set as loose safety bounds rather than tight physical motion limits. These values are used only to bound the LiDAR-only motion prior during deskewing, not to constrain the final pose estimated by registration. Therefore, they do not directly limit the actual terrain-induced elevation change estimated by ICP. The purpose of the clamping operation is not to suppress normal gait-induced vertical motion or slope-induced attitude changes, but to prevent extreme and physically implausible spikes in the LiDAR-only motion prior from being propagated into the deskewing process.

For other robots or gaits, the same principle can be applied by selecting the thresholds according to the expected intra-scan vertical and angular motion range of the target platform. As a practical guideline, $\zeta_{z}$ can be initially set based on the maximum expected vertical displacement within one LiDAR scan period, e.g., the product of the platform’s maximum climbing speed and the LiDAR scan period, while $\zeta_{r}$ and $\zeta_{p}$ can be selected according to the maximum expected *roll* and *pitch* variations within a single scan. These bounds should be large enough to preserve genuine terrain-induced motion, but small enough to reject clearly abnormal motion-prior spikes. The corrected end pose is then reconstructed as

(7) $${\tilde{T}}_{f}=T_{s}exp(\tilde{\xi }).$$

The scan is then partitioned into multiple temporal segments, and the pose at each segment boundary $T_{k}$ is computed by interpolating between the start pose $T_{s}$ and the corrected end pose ${\tilde{T}}_{f}$ using SLERP for rotation and linear interpolation for translation:

(8) $$T_{k}=I\left(T_{s},\;{\tilde{T}}_{f},\;\frac{k}{K}\right),\;k=0,\ldots ,K,$$

where I(⋅) denotes the combined rotation-translation interpolation operator.

(9) $$\xi_{k}=log(T_{k}^{-1}T_{k+1}),\;k=0,\ldots ,K-1.$$

Rather than using a single twist for the entire scan, a separate local twist is permitted for each segment, enabling a better approximation of the temporal variation of intra-scan motion. Let $\tau_{i}\in [0,1]$ denote the normalized acquisition time of the *i*-th point, obtained from its LiDAR timestamp, and let $\tau_{k}=k/K$ denote the normalized boundary time of the *k*-th temporal segment. For each point, the segment index *k* is selected such that $\tau_{k}\leq \tau_{i}<\tau_{k+1}$. The intra-segment interpolation coefficient is then computed as

(10) $$u_{i}=\frac{\tau_{i}-\tau_{k}}{\tau_{k+1}-\tau_{k}},\;\tau_{k}=\frac{k}{K}.$$

The sensor pose at the corresponding time instant is computed as

(11) $$T(\tau_{i})=T_{k}exp(u_{i}\xi_{k}).$$

The mid-scan pose is used as the reference frame, and all points are corrected to this common coordinate system:

(12) $$T_{mid}=T(0.5),\;p_{i}^{*}=T_{mid}^{-1}\;T(\tau_{i})\;p_{i}.$$

The mid-scan instant is chosen as the reference because, compared to using the start or end of the scan, it distributes the correction deviations symmetrically along the time axis, thereby reducing the maximum per-point correction error. Consequently, even in the presence of prediction error, the accumulation of deskewing error due to reference-time bias is mitigated.

In summary, the proposed deskewing approximates the nonlinear intra-scan motion on a per-segment basis while simultaneously suppressing instantaneous vertical spikes and abrupt *roll*/*pitch* changes—which occur frequently during quadrupedal locomotion—through clamping. As shown in Figure 1, the resulting corrected point cloud serves as the input to the decoupled two-stage registration proposed in Section 3.3.

### 3.3. Horizontal–Vertical Decoupled Two-Stage Registration

Taking the point cloud corrected in Section 3.2 as input, the proposed method performs two registration stages sequentially at each outer ICP iteration. First, horizontal registration based on point-to-point correspondences estimates the planar motion components $\left(x,y,\psi \right)$. Then, vertical registration based on point-to-plane correspondences refines the elevation-related components $\left(z,\phi ,\theta \right)$. This section describes the residual, Jacobian, and state update structure of each stage, while the correspondence weighting and update stabilization strategy for the vertical stage are detailed in Section 3.4.

Point-to-point residuals are used in the horizontal stage because the horizontal motion components $\left(x,y,\psi \right)$ are generally well constrained by the spatial distribution of surrounding LiDAR points. This choice preserves the robustness and simplicity of the KISS-ICP baseline and avoids relying on potentially noisy normal estimates during the primary horizontal alignment. Although point-to-plane residuals may provide stronger directional constraints, their performance depends heavily on the quality of local normal estimation. Under quadruped locomotion, gait-induced vibration, sparse LiDAR measurements, and residual motion distortion can make local normals noisy or inconsistent, which may introduce biased constraints into horizontal estimation.

In contrast, the vertical components $\left(z,\phi ,\theta \right)$ are more susceptible to weak observability in elevation-varying environments. Therefore, point-to-plane residuals are introduced only in the vertical refinement stage, where surface normals provide useful directional constraints for suppressing *Z*-axis drift and stabilizing attitude estimation.

#### 3.3.1. Horizontal Stage: Planar Motion Estimation via Point-to-Point Constraints

The horizontal stage estimates only *x*, *y*, and *yaw* using point-to-point residuals. Letting $s_{i}$ and $q_{i}$ denote the current source point and its corresponding target point, respectively, the residual is defined as

(13) $$r_{i}^{H}=s_{i}-q_{i}.$$

The full 6-DoF Jacobian is given by

(14) $$J_{i}^{p}=\left[\begin{array}{cc} I_{3\times 3} & -[s_{i}{]}_{\times } \end{array}\right]\in R^{3\times 6},$$

where $[s_{i}{]}_{\times }$ denotes the skew-symmetric matrix. Since the horizontal stage estimates only $\delta \xi_{H}=[\delta x,\delta y,\delta \psi {]}^{⊤}$, only the columns of the full Jacobian corresponding to x, y, and yaw are used. Defining the state selection matrix as

(15) $$S_{H}=\left[\begin{array}{ccc} 1 & 0 & 0\\ 0 & 1 & 0\\ 0 & 0 & 0\\ 0 & 0 & 0\\ 0 & 0 & 0\\ 0 & 0 & 1 \end{array}\right]\in R^{6\times 3},$$

the reduced Jacobian is expressed as

(16) $${\bar{J}}_{i}^{H}=J_{i}^{p}S_{H}\in R^{3\times 3}.$$

Conceptually, the normal equation of the horizontal stage is formulated over $\delta \xi_{H}\in R^{3}$; in the implementation, this is equivalently realized as a masked solve that sets the inactive components of the full 6-DoF update to zero. Written in full-state form, the horizontal-stage update is

(17) $${\hat{\delta \xi }}_{H}={\left[\begin{array}{cccccc} \delta x & \delta y & 0 & 0 & 0 & \delta \psi \end{array}\right]}^{⊤}.$$

The Hessian and gradient of the horizontal stage are computed as

(18) $$H_{H}=\sum_{i}w_{i}^{H}{{\bar{J}}_{i}^{H}}^{⊤}{\bar{J}}_{i}^{H},g_{H}=\sum_{i}w_{i}^{H}{{\bar{J}}_{i}^{H}}^{⊤}r_{i}^{H},$$

where $w_{i}^{H}$ is a squared Lorentzian robust weight defined by the residual magnitude, formulated as

(19) $$w_{i}^{H}=\frac{\kappa_{H}^{2}}{{\left(\kappa_{H}+{∥r_{i}^{H}∥}_{2}^{2}\right)}^{2}},$$

where $\kappa_{H}$ is a robust kernel scale parameter that controls the attenuation of large-residual correspondences. This weight monotonically decreases with residual magnitude, suppressing the influence of large-residual correspondences while preserving the contribution of well-matched ones.

To prevent convergence failure due to insufficient correspondences in the early iterations, the horizontal stage applies an iteration-dependent correspondence distance. For a base maximum correspondence distance $d_{max}$, the search radius at the t-th outer ICP iteration is defined as

(20) $$d_{max}(t)=\left\{\begin{array}{cc} d_{relaxed}, & t\in T_{early}\\ d_{max}, & otherwise \end{array}\right.$$

where $d_{relaxed}$ denotes a temporarily relaxed correspondence distance, $d_{max}$ is the nominal maximum correspondence distance, and $T_{early}$ denotes the set of early ICP iterations. Afterward, the search radius returns to the nominal value to suppress excessive false correspondences. The update ${\hat{\delta \xi }}_{H}$ computed in the horizontal stage is applied to the current estimated pose, aligning only the planar motion while keeping vertical translation and *roll*/*pitch* fixed.

#### 3.3.2. Vertical Stage: Attitude Refinement via Point-to-Plane Constraints

After the horizontal state has been corrected, the vertical state $\left(z,\phi ,\theta \right)$ is refined in a separate stage. Because the vertical direction is difficult to observe stably from point-to-point residuals alone, point-to-plane residuals are used to exploit the structural geometry of ground surfaces and sloped structures in elevation-varying environments.

First, the local surface normal $n_{i}$ is estimated around each source point $s_{i}$. Neighboring points are collected from adjacent voxels in the target map, and the mean ${\bar{p}}_{i}$ and covariance matrix of those points are computed:

(21) $$C_{i}=\frac{1}{|N_{i}|}\sum_{p\in N_{i}}(p-{\bar{p}}_{i}){\left(p-{\bar{p}}_{i}\right)}^{⊤},$$

where $N_{i}$ is the local neighborhood of $s_{i}$. The eigenvector corresponding to the smallest eigenvalue of the covariance matrix is used as the normal:

(22) $$n_{i}=v_{min}(C_{i}).$$

Not all points are valid for vertical state estimation. To avoid unstable vertical updates, a point is admitted as a vertical constraint only when its local neighborhood, normal orientation, and range satisfy predefined reliability conditions.

The point-to-plane residual for a selected correspondence is defined as

(23) $$r_{i}^{V}=n_{i}^{⊤}(s_{i}-q_{i}),$$

where $q_{i}$ is the nearest target point selected within the local voxel neighborhood. The full 6-DoF Jacobian is

(24) $$J_{i}^{V}=\left[\begin{array}{cc} n_{i}^{⊤} & n_{i}^{⊤}(-[s_{i}{]}_{\times }) \end{array}\right]\in R^{1\times 6}.$$

Only the columns corresponding to z, *roll*, and *pitch* are used in the vertical stage. Defining the state selection matrix as

(25) $$S_{V}=\left[\begin{array}{ccc} 0 & 0 & 0\\ 0 & 0 & 0\\ 1 & 0 & 0\\ 0 & 1 & 0\\ 0 & 0 & 1\\ 0 & 0 & 0 \end{array}\right]\in R^{6\times 3},$$

the reduced Jacobian is

(26) $${\bar{J}}_{i}^{V}=J_{i}^{V}S_{V}\in R^{1\times 3}.$$

The normal equation of the vertical stage is therefore formulated over $\delta \xi_{V}=[\delta z,\delta \phi ,\delta \theta {]}^{⊤}\in R^{3}$; the definition of the correspondence weight $w_{i}^{V}$ and the explicit form of the resulting Hessian and gradient are given in Section 3.4. Written in full-state form, the vertical-stage update is

(27) $${\hat{\delta \xi }}_{V}={\left[\begin{array}{cccccc} 0 & 0 & \delta z & \delta \phi & \delta \theta & 0 \end{array}\right]}^{⊤}.$$

That is, x, y, and yaw—already aligned in the horizontal stage—are preserved, while only vertical translation and *roll*/*pitch* are refined to conform to the terrain geometry. In the implementation, the vertical stage may be iterated multiple times within each outer ICP iteration to improve convergence of the vertical state.

Additionally, the vertical stage applies a per-correspondence reliability weight and a bounded update strategy to suppress excessive vertical corrections.

### 3.4. Observability-Aware Vertical State Stabilization

This stabilization process corresponds to the observability-aware weighting and bounded vertical update components in the two-stage ICP module of Figure 1. The quality of vertical state estimation depends not simply on the magnitude of residuals, but on how well each correspondence actually constrains *z*, *roll*, and *pitch*. For instance, a slope point observed at close range can strongly explain the vertical state, whereas a sparse point at long range or a nearly vertical wall point carries significantly less information about the vertical state. Treating all correspondences equally therefore causes the vertical optimization to easily become unstable, unable to distinguish between informative terrain observations and unreliable ones.

To address this, the proposed method formalizes the geometric validity of each correspondence as an observability score and combines it with a residual-based robust weight to form the final weight for the vertical stage. First, the distance-based observability term is defined as

(28) $$\gamma (d_{i})=\left\{\begin{array}{cc} 1, & d_{i}<d_{0},\\ \frac{1}{1+\beta (d_{i}-d_{0}{)}^{2}}, & d_{i}\geq d_{0}, \end{array}\right.$$

where $d_{i}=∥s_{i}-o∥$ is the distance from the sensor origin o to source point $s_{i}$. This function maintains maximum confidence for near-range points while progressively reducing the contribution of far-range points as distance increases, where $d_{0}$ and β control the transition point and decay rate of the distance-based observability term, respectively.

Next, to reflect how well the surface orientation constrains the vertical state, the normal-based observability term is defined as

(29) $$\eta (n_{i})=|n_{i,z}|.$$

A larger z-component of the normal indicates that the surface is closer to horizontal (ground or slope), providing more meaningful information for estimating z, *roll*, and *pitch*. Conversely, when $|n_{i,z}|$ is small—as for a nearly vertical wall—the surface poorly constrains the vertical state, and its influence is accordingly reduced. The proposed observability score is designed as a practical geometry-based surrogate indicator rather than a formal probabilistic observability metric. Although a Fisher-information-based formulation could provide a stronger theoretical foundation, the proposed distance- and normal-based terms provide a computationally efficient approximation for evaluating the reliability of vertical point-to-plane constraints. This design is consistent with the geometric and optimization-structure-based reasoning used in existing degeneracy- and localizability-aware registration methods, which identify weakly constrained directions based on local geometric structure, Hessian eigenspace, Jacobian contribution, or surface-normal alignment.

Combining these two factors, the observability score of correspondence i is defined as

(30) $$\Omega_{i}=\gamma (d_{i})\;\eta (n_{i}).$$

However, high observability does not justify blindly incorporating residual outliers. Therefore, a robust term $\rho_{i}$ based on residual magnitude is additionally introduced. In particular, near-range points may carry important evidence of actual vertical change even when they produce large residuals upon entering a steep slope; rather than applying a uniform continuous kernel that would attenuate all such responses, the proposed piecewise design aggressively follows near-range observations and suppresses only clear mismatches. Accordingly, $\rho_{i}$ is defined as

(31) $$\rho_{i}=\left\{\begin{array}{cc} 1, & d_{i}<d_{0}\;\mathrm{and}\;|r_{i}^{V}|\;\leq \epsilon_{0},\\ \epsilon_{s}, & d_{i}<d_{0}\;\mathrm{and}\;|r_{i}^{V}|\;>\epsilon_{0},\\ \frac{\kappa_{V}^{2}}{\kappa_{V}^{2}+(r_{i}^{V}{)}^{2}}, & d_{i}\geq d_{0}, \end{array}\right.$$

where $r_{i}^{V}$ is the point-to-plane residual defined in Section 3.3.2, and $\epsilon_{0}$, $\epsilon_{s}$, and $\kappa_{V}$ denote the residual gating threshold, the minimum suppression weight, and the robust kernel scale parameter, respectively. The discontinuous transition in the near-range regime is intentional: comparatively large residuals that arise near a steep slope or stair entry are accepted as is so that the vertical state can respond rapidly to terrain change, while only sufficiently large residuals in the near-range regime are immediately suppressed to their minimum weight $\epsilon_{s}$ to block the influence of clear mismatches. In the far-range regime, where measurement uncertainty is relatively high, a Cauchy-type continuous kernel is used to progressively reduce the weight as the residual grows. To prevent this correspondence-level discontinuity from causing unstable state updates, the trust-region bound introduced in Equation (34) limits the norm of the vertical update vector. Thus, the robust weight and the trust-region bound work together as a two-level stabilization mechanism: correspondence-level outlier suppression and state-level update limiting.

The final correspondence weight for the vertical stage is defined as

(32) $$w_{i}^{V}=\rho_{i}\;\Omega_{i}.$$

Using the reduced Jacobian ${\bar{J}}_{i}^{V}$ defined in Section 3.3.2, the Hessian and gradient of the vertical stage are then computed as

(33) $$H_{V}=\sum_{i}w_{i}^{V}{{\bar{J}}_{i}^{V}}^{⊤}{\bar{J}}_{i}^{V},g_{V}=\sum_{i}w_{i}^{V}{{\bar{J}}_{i}^{V}}^{⊤}r_{i}^{V}.$$

Correspondences with low observability scores automatically contribute less to the Hessian, so the vertical update is shaped primarily by geometrically informative terrain structures. In this sense, the observability score in the proposed method is not merely a quality indicator but serves to regulate the effective information content of the optimization.

In addition, a trust-region-based update limit is introduced to prevent excessive vertical corrections. Let ${\tilde{\delta \xi }}_{V}$ denote the vertical update obtained by solving the normal equation; the final update ${\hat{\delta \xi }}_{V}$ is determined as

(34) $${\hat{\delta \xi }}_{V}=\left\{\begin{array}{cc} {\tilde{\delta \xi }}_{V}, & {∥{\tilde{\delta \xi }}_{V}∥}_{2}\leq \zeta_{V},\\ \zeta_{V}\frac{{\tilde{\delta \xi }}_{V}}{{∥{\tilde{\delta \xi }}_{V}∥}_{2}}, & ∥{\tilde{\delta \xi }}_{V}{∥}_{2}>\zeta_{V}. \end{array}\right.$$

When the constraint is violated, rather than discarding the update or clipping individual components, the direction of the full vertical update vector is preserved and only its norm is normalized to $\zeta_{V}$, where $\zeta_{V}$ denotes the trust-region bound for the vertical state update. By directly limiting the magnitude of the se(3) update vector, this suppresses excessive correction in a single iteration even when a momentarily large residual forms at an abrupt elevation transition or steep slope entry. In other words, while observability-aware weighting regulates information reliability at the correspondence level, the trust region guarantees stability at the state update level.

These weighting and trust-region constraints are recomputed and applied at every vertical sub-iteration described in Section 3.3.2. Consequently, the vertical stage operates not by performing a large correction at once, but by iteratively accumulating bounded updates while prioritizing correspondences with high observability. This is advantageous for gradually aligning the vertical state while preserving the horizontal pose in environments with varying elevation.

By combining correspondence-level weighting with state-level update limiting, the proposed method can handle the substantially terrain-dependent observability of the vertical state in a more stable manner. The following section summarizes the complete estimation procedure incorporating the deskewing, state decomposition, and vertical stabilization strategies described above.

### 3.5. Complete Estimation Procedure

The complete estimation procedure of the proposed front-end is summarized in Algorithm 1 and illustrated in Figure 1. The core of the proposed front-end lies in integrating the deskewing, state decomposition, and vertical stabilization strategies described in the preceding sections into a single sequential estimation procedure.

First, Piecewise-Constant Velocity deskewing is performed using a motion prior constructed from the predicted start and end poses of the current scan. *Z*-axis motion clamping is applied during this process to suppress abnormal vertical oscillations and abrupt *roll*/*pitch* changes from being excessively reflected in the initial correction. The purpose of this stage is not merely to reduce scan distortion but to stabilize the input to registration itself, so that subsequent correspondence formation can take place on a more geometrically consistent foundation.

Because the deskewed point cloud is expressed in the mid-scan reference frame, the initial registration estimate $T_{0}$ is set to the predicted mid-scan pose derived from the same motion prior used for deskewing. That is, $T_{0}$ is the initial alignment pose consistent with the reference frame of Section 3.2, and from an algorithmic perspective it represents the prior pose that ICP is tasked with correcting. ICP then operates by accumulating an incremental correction $T_{icp}$ on top of this initial estimate, and the final pose is recovered as $T_{k}=T_{icp}\;T_{0}$.

Outer ICP iterations are then performed on the deskewed point cloud. In each iteration, the horizontal stage first executes, aligning x, y, and yaw via point-to-point constraints. During the early ICP iterations, the correspondence distance is temporarily relaxed to prevent a shortage of correspondences caused by abrupt elevation changes or initial deskewing errors. Once the horizontal stage has primarily aligned the planar motion of the current frame, the vertical stage refines only z, *roll*, and *pitch* via point-to-plane constraints while holding the already-aligned horizontal state fixed. The geometric validity of each correspondence is reflected through observability-aware weighting, and the computed vertical update is applied after passing through a trust-region limit. The vertical stage may also be iterated multiple times within a single outer iteration to progressively correct the vertical pose, which typically converges more slowly than the horizontal pose in environments with varying elevation. As a result, horizontal registration first stabilizes the reference coordinate frame, and vertical registration then iteratively accumulates bounded updates on top of it, allowing the odometry front-end to respond without unstable vertical observations contaminating the horizontal trajectory.

Upon termination of the outer ICP iterations, the aligned scan is integrated into the local map and the updated pose is used as the motion prior and initial registration estimate for the next frame. Each frame is processed through a fixed sequence in which motion compensation first stabilizes the geometric input, horizontal registration then establishes a reliable planar reference, and vertical refinement subsequently adjusts height and attitude within that reference before the aligned scan is incorporated into the map.
**Algorithm 1.** Proposed Front-End Estimation ProcedureInputCurrent scan $P={\left\{(p_{i},\tau_{i})\right\}}^{ni=1}$Predicted start pose $T_{s}$ and end pose $T_{f}$Local map MOutputUpdated pose $T_{k}$Updated local map M1.Compute relative motion: $\Delta T\leftarrow {T_{s}}^{-1}\;T_{f},\;\xi \leftarrow log\left(\Delta T\right)$
2.Construct corrected twist by clamping vertical translation and attitude com-ponents:
$\tilde{\xi }\leftarrow clamp\left(\xi \right)$
3.Reconstruct corrected end pose:
${\tilde{T}}_{f}\leftarrow T_{s}\;exp\left(\tilde{\xi }\right)$
4.Partition the scan into K temporal segments.5.Interpolate segment boundary poses:
$T_{k}\leftarrow I\left(T_{s},\;T_{f},\;\frac{k}{K}\right),\;k=0,\;\ldots ,\;K$
6.Compute local twist for each segment: $\xi_{k}\leftarrow log\left({T_{k}}^{-1}\;T_{k+1}\right),\;k=0,\;\ldots ,\;K-1$7.For each point $p_{i}$, compute its deskewed position ${p_{i}}^{*}$ using the corresponding segment pose interpolation.8.Set the mid-scan pose as the initial registration estimate: $T_{0}\leftarrow T\left(0.5\right)$
9.Initialize ICP correction:
$T_{icp}\leftarrow I$
10.for each outer ICP iteration t do11.  if $t\in T_{early}$ then12.    $d_{corr}\left(t\right)\leftarrow d_{relaxed}$
13.  else14.    $d_{corr}\left(t\right)\leftarrow d_{max}$
15.  end if16.  Find correspondences for the deskewed scan using
$d_{corr}\left(t\right).$
17.  Estimate horizontal update $\delta \xi_{H}$ using point-to-point residuals over (x, y, yaw).18.  Apply the horizontal update:
$T_{icp}\leftarrow exp\left(\delta {\tilde{\xi }}_{H}\right)\cdot T_{icp}$
19.  for $u=1,\;\ldots ,\;U_{V}$ do20.    Estimate local normals for candidate correspondences.21.    Filter valid vertical correspondences satisfying:     $\left|N_{i}\right|\geq N_{min},\;\left|n_{i,z}\right|\geq \tau_{n},\;\left\|s_{i}-o\right\|\leq r_{max}$22.    Compute observability-aware vertical weights
$w_{i}^{V}$
23.    Estimate vertical update $\delta \xi_{V}$ using point-to-plane residuals over (z, roll, pitch).24.    if ${\left\|\delta \xi_{V}\right\|}_{2}>\zeta_{V}$ then25.      
$\delta \xi_{V}\leftarrow \frac{\zeta_{V}\cdot \delta \xi_{V}}{{\left\|\delta \xi_{V}\right\|}_{2}}$
26.    end if27.    Apply the vertical update:
$T_{icp}\leftarrow exp\left(\delta {\tilde{\xi }}_{V}\right)\cdot T_{icp}$
28.  end for29.end for30.Recover final pose:
$T_{k}\leftarrow T_{icp}\cdot T_{0}$
31.Integrate the aligned scan into the local map
M.
32.Return
$T_{k}$ and updated
M.


## 4. Experiments

This section evaluates the proposed LiDAR odometry method both quantitatively and qualitatively. We first describe the datasets, compared methods, implementation details, and evaluation protocol. We then present the main comparison against representative baselines, followed by qualitative trajectory analysis.

### 4.1. Experimental Setup

#### 4.1.1. Datasets and Sequences

**Figure 3:**
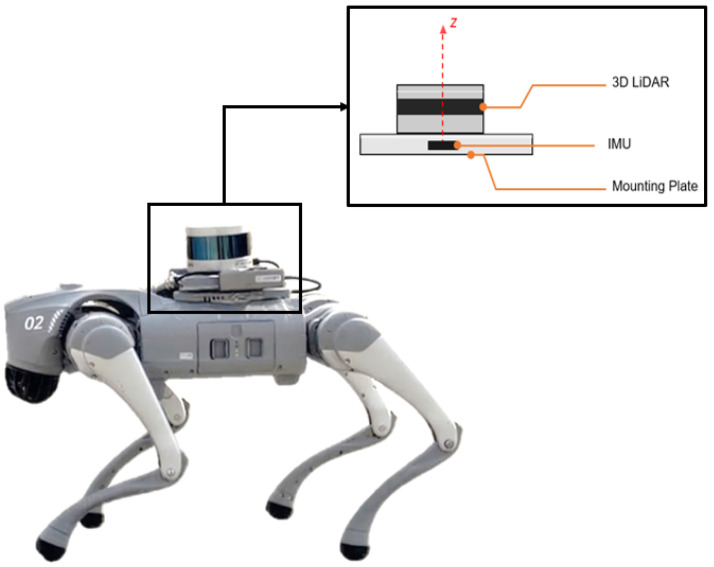
Experimental platform: Unitree Go2 quadruped robot equipped with LiDAR and IMU.

**Figure 4:**
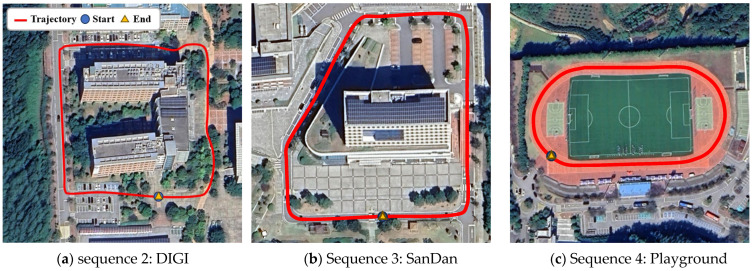
Satellite view of the data collection environments for the quadruped robot sequences.

We evaluated the proposed method on four sequences. Sequence 1 is the Playground sequence from the public S3E dataset [33], acquired using a wheeled robot platform with a Velodyne VLP-16 Puck LiDAR (Velodyne Lidar Inc., San Jose, CA, USA). Sequences 2–4 were collected using a Unitree Go2 quadruped robot equipped with a Velodyne VLP-16 LiDAR. The experimental platform and sensor configuration are shown in Figure 3. Sequence 2 (DIGI) and Sequence 3 (SanDan) include ramps, whereas Sequence 1 (S3E Playground) and Sequence 4 (Go2 Playground) do not contain sloped segments. The data collection environments for the quadruped robot sequences are illustrated in Figure 4. An overview of the sequences is provided in Table 1.

The four sequences were selected to separate the effects of platform motion and terrain elevation on LiDAR odometry. Sequence 1 was included as a public wheeled-robot sequence with dataset-provided ground truth and relatively smooth platform motion, providing a standard reference case. Sequences 2 and 3 were selected as quadruped-robot sequences containing ramps or slopes, where gait-induced vibration, abrupt *roll*/*pitch* variation, and elevation changes jointly challenge LiDAR odometry. Sequence 4 was selected as a flat-terrain quadruped sequence to examine whether the proposed method also improves odometry under quadruped locomotion even without explicit elevation changes. This scenario design allows the evaluation to cover representative conditions for mobile robot navigation, including smooth wheeled motion, elevation-varying quadruped locomotion, and flat-terrain quadruped locomotion.

All experiments were conducted on the same hardware platform consisting of a 12th Gen Intel Core i7-12700F CPU, 16 GB RAM, and an NVIDIA GeForce RTX 3070 GPU. All compared methods were evaluated on identical input point-cloud sequences, and the GPU was additionally used for pseudo-ground-truth generation with GLIM [34].

#### 4.1.2. Compared Methods and Reference Trajectory

The proposed method was compared against three representative LiDAR registration and odometry methods: ICP [8], KISS-ICP [7], and F-LOAM [5]. ICP represents classical raw point-cloud registration, KISS-ICP is a lightweight point-based LiDAR odometry method, and F-LOAM represents a feature-based LiDAR odometry pipeline. These methods provide a meaningful comparison across geometry-only registration, lightweight point-based odometry, and feature-based odometry.

The reference trajectory differed depending on the dataset source. For Sequence 1 (S3E Playground), we used the ground-truth trajectory provided by the public dataset; therefore, the errors on this sequence represent evaluation against real dataset-provided ground truth. For Sequences 2–4, which were collected using the Unitree Go2 quadruped robot in outdoor environments, externally measured ground truth such as motion capture, RTK-GNSS, or total-station measurements was not available. Therefore, we used GLIM-generated trajectories as pseudo ground truth (pGT). GLIM estimates the trajectory offline using LiDAR and iAHRS IMU measurements with GPU-accelerated scan-matching factors and fixed-lag smoothing, providing a consistent high-quality reference for relative comparison. On Sequence 1, GLIM also achieved the lowest error among all compared methods against the publicly provided ground truth, which further supports its use as a reference trajectory generator for Sequences 2–4. Nevertheless, this pGT is not an independently measured absolute ground truth. Accordingly, the errors reported for Sequences 2–4 should be interpreted as relative comparisons against a consistent reference trajectory, rather than as strict absolute-error bounds.

Performance was evaluated using Absolute Pose Error (APE). For each sequence, we report the maximum, mean, RMSE, and standard deviation of APE. RMSE and mean APE reflect overall trajectory accuracy, while the maximum error and standard deviation indicate worst-case behavior and estimation stability, respectively. All results were computed using the evo toolkit [35].

### 4.2. Comparison with State of the Arts

Table 2 and Table 3 summarize the detailed quantitative results in terms of mean APE and RMSE across all four sequences. The best results are shown in bold, and the second-best results are underlined.

On Sequence 1 (S3E Playground, wheeled robot), the proposed method reduced the mean APE from 4.52 m to 1.75 m and the RMSE from 5.38 m to 2.11 m relative to KISS-ICP. This result indicates that the proposed deskewing and registration strategy remains beneficial even under comparatively smooth wheeled-motion conditions.

The improvements become substantially larger on the quadruped sequences. On Sequence 2 (DIGI), the proposed method achieved a mean APE of 1.34 m and an RMSE of 1.71 m, whereas KISS-ICP yielded 15.48 m and 20.94 m, respectively. This large gap suggests that the baseline formulation is more vulnerable to high-frequency body motion and abrupt attitude variation in quadruped locomotion.

A similar trend appears on Sequence 3 (SanDan), which also contains sloped segments. The proposed method obtained a mean APE of 2.85 m and an RMSE of 3.71 m, compared with 12.02 m and 15.32 m for KISS-ICP. Although F-LOAM performed better than KISS-ICP on this sequence, it still remained clearly behind the proposed method, indicating that the proposed point-based framework can remain competitive without relying on edge- and plane-feature extraction.

On Sequence 4 (Go2 Playground, quadruped, no slope), the proposed method again achieved the lowest error, with a mean APE of 3.89 m and an RMSE of 4.93 m. In contrast, KISS-ICP produced a mean APE of 11.95 m and an RMSE of 13.96 m, while F-LOAM showed the largest degradation. This result indicates that the benefit of the proposed method is not limited to ramp handling, but also extends to general vibration-induced distortion and vertical inconsistency during quadruped motion.

Overall, the proposed method consistently achieved the lowest error among the compared odometry baselines across all four sequences.

### 4.3. Qualitative Results

**Figure 5:**
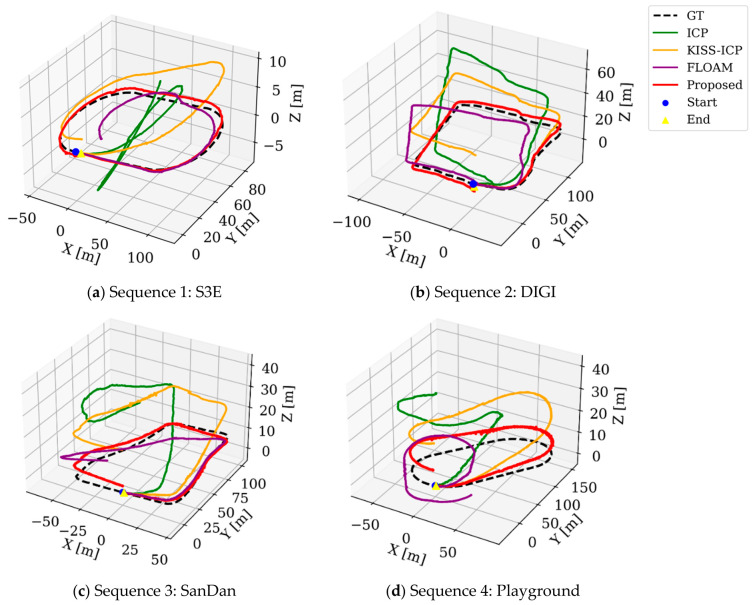
Three-dimensional trajectory comparison across all four sequences.

Figure 5 presents three-dimensional trajectory comparisons for all four sequences. On Sequence 1 (S3E, wheeled robot), the baseline methods already show noticeable three-dimensional inconsistency with respect to the reference trajectory. ICP exhibits severe spatial distortion with large vertical excursions, KISS-ICP preserves the loop tendency but still shows clear *Z*-axis deviation, and F-LOAM forms a contracted inner loop rather than following the reference boundary. In contrast, the proposed method remains most closely aligned with the reference trajectory in all three axes. On Sequence 2 (DIGI), which includes ramps, ICP and F-LOAM diverge substantially from the reference trajectory, while KISS-ICP preserves the overall path geometry but exhibits significant vertical drift on the sloped segment. In contrast, the proposed method remains tightly aligned with the reference throughout the sequence. A similar trend is observed on Sequence 3 (SanDan), where the baseline methods exhibit varying degrees of three-dimensional drift, particularly in the vertical direction, whereas the proposed method maintains a trajectory shape most consistent with the reference. On Sequence 4 (Go2 Playground), which does not contain ramps, ICP and F-LOAM still show large spatial deviations caused by quadruped locomotion, while KISS-ICP preserves the overall loop shape more successfully but accumulates noticeable drift over the full trajectory. The proposed method again remains closest to the reference, preserving the loop geometry most faithfully despite vibration-induced motion distortion. Across all sequences, the proposed method produces the most consistent three-dimensional trajectory, with particularly clear advantages in vertical stability on the elevation-varying sequences.

**Figure 6:**
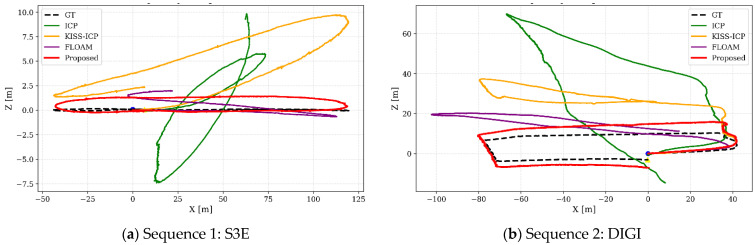

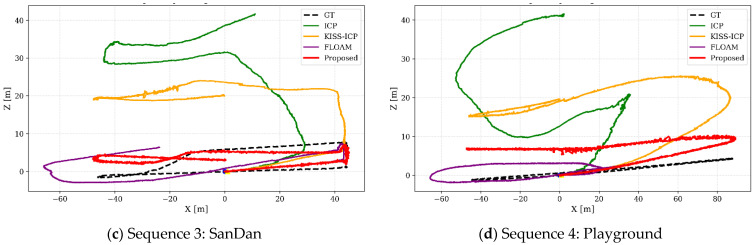
Side-view trajectory comparison illustrating vertical drift suppression across all four sequences. The blue circle indicates the start position, and the yellow triangle indicates the end position.

**Figure 7:**
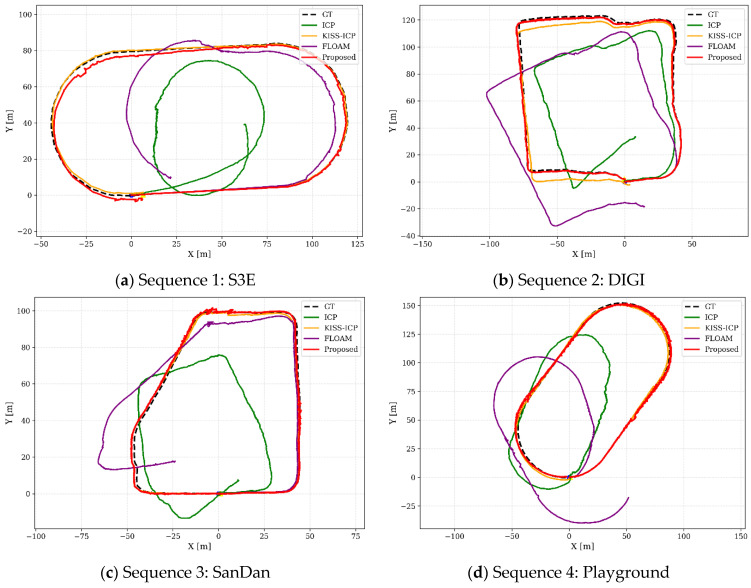
Top-down view trajectory comparison. The blue circle indicates the start position, and the yellow triangle indicates the end position.

Figure 6 presents side-view trajectory comparisons in the X–Z plane, directly illustrating the vertical drift behavior of each method. On Sequence 1, ICP produces large negative and positive Z excursions, and KISS-ICP also deviates noticeably upward from the near-flat reference profile. F-LOAM remains relatively bounded in the vertical direction, although it still shows a visible offset from the reference. In contrast, the proposed method remains closely aligned with the reference trajectory and maintains the most stable near-zero height profile throughout. On Sequence 2 (DIGI), ICP and KISS-ICP exhibit severe upward Z drift upon entering the ramp, while F-LOAM also shows a clear elevation mismatch and larger vertical error than the proposed method. The proposed method tracks the gradual elevation change most smoothly without unstable vertical jumps. A similar pattern is observed on Sequence 3 (SanDan), which contains a longer sloped segment: ICP and KISS-ICP exhibit pronounced vertical drift, whereas F-LOAM shows relatively limited *Z*-axis divergence but still does not follow the reference slope profile as closely as the proposed method. On Sequence 4 (Playground), even without ramps, ICP and KISS-ICP show considerable vertical oscillation and drift caused by quadruped locomotion alone. F-LOAM again remains relatively bounded in Z, but the proposed method stays closest to the flat reference trajectory and suppresses vertical oscillations most effectively. Overall, F-LOAM does not exhibit the largest vertical error in most sequences and tends to remain more bounded in Z than the point-based baselines; however, its reference consistency remains limited, and its large horizontal distortion, shown in Figure 5 and Figure 7, still degrades overall trajectory accuracy. These results indicate that the decoupled horizontal–vertical optimization and observability-aware vertical refinement effectively reduce *Z*-axis drift across both flat and elevation-varying terrain conditions.

Figure 7 presents top-down trajectory comparisons in the X–Y plane. On Sequence 1, the proposed method and KISS-ICP follow the outer loop shape relatively closely, while ICP forms a contracted inner loop and F-LOAM shows a laterally shifted trajectory with a different loop shape. On Sequence 2 (DIGI), the proposed method remains closest to the reference boundary. KISS-ICP preserves the overall rectangular loop shape but shows a visible offset and scale difference, whereas ICP and F-LOAM deviate substantially from the reference path. On Sequence 3 (SanDan), the proposed method and KISS-ICP again follow the reference loop closely, while ICP produces a contracted inner trajectory and F-LOAM shows a distorted path, particularly on the left side of the loop. On Sequence 4 (Playground), the proposed method and KISS-ICP both preserve the overall loop shape, with the proposed method remaining closest to the reference trajectory. In contrast, ICP forms an inner distorted loop and F-LOAM shows a largely deformed trajectory. Overall, the proposed method remains closest to the reference across all four sequences, while KISS-ICP also shows relatively good horizontal agreement in the X–Y plane.

### 4.4. Further Analysis and Discussion

A key observation from the experiments is that the proposed method yields much larger gains on the quadruped sequences than on the wheeled sequence. KISS-ICP relies on a constant-velocity motion model for deskewing and a coupled point-to-point registration framework. Such assumptions are adequate when the platform motion within a LiDAR sweep is small and smooth, but become less reliable under rapid vertical oscillation, abrupt *roll*/*pitch* changes, and irregular body motion, as is common in quadruped locomotion.

The improvements are particularly pronounced on DIGI and SanDan, both of which contain ramps. This suggests that the main advantage of the proposed method is not only more accurate deskewing but also more stable handling of the vertical degrees of freedom. In standard 6-DoF coupled optimization, unstable updates in *z*, *roll*, and *pitch* can contaminate the horizontal estimate. The proposed method mitigates this issue by separating horizontal and vertical updates and introducing an additional vertical refinement process with point-to-plane constraints.

Another notable result is that the advantage of the proposed method remains visible even on Sequence 4, which contains no ramps. This indicates that the proposed design addresses not only slope-induced instability but also the broader scan distortion and vertical inconsistency caused by quadruped locomotion itself. The method can therefore be understood as a LiDAR odometry design tailored to the motion characteristics of legged robots rather than a terrain-specific correction alone.

**Figure 8:**
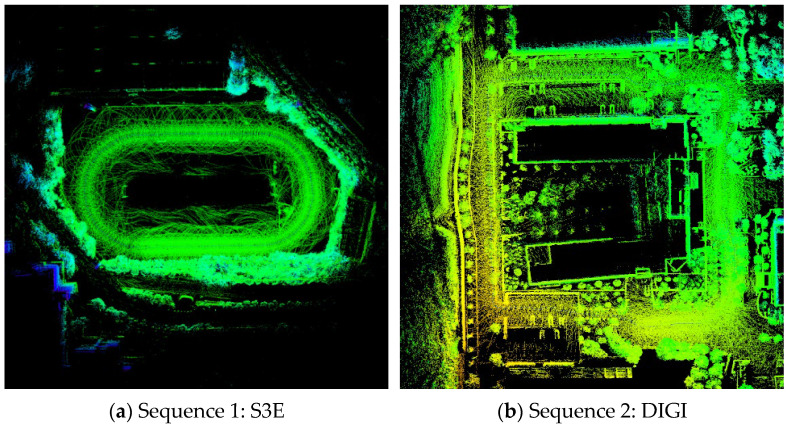

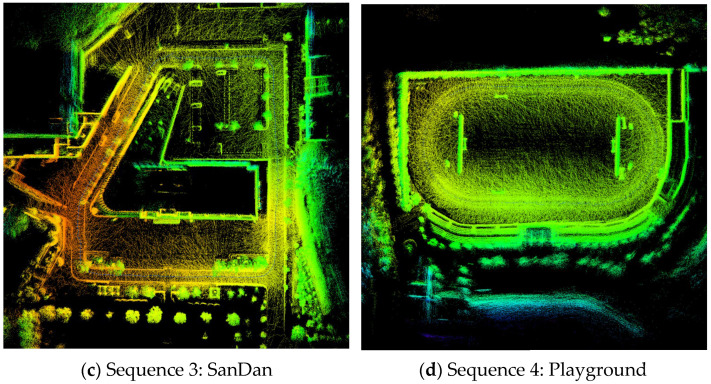
Point-cloud maps generated by the proposed odometry front-end with Scan Context-based loop closure. The color map represents height information, with cooler colors indicating lower elevations and warmer colors indicating higher elevations.

Figure 8 shows point-cloud maps generated by integrating the proposed odometry front-end with Scan Context [36]-based loop closure and pose graph optimization, demonstrating that the proposed front-end is compatible with standard back-end components without architectural modification.

This study also has several limitations. First, the quadruped sequences were evaluated against GLIM-based pseudo ground truth rather than externally measured absolute ground truth. Although Sequence 1 was evaluated using the ground-truth trajectory provided by the public S3E dataset, Sequences 2–4 did not include external ground truth from motion capture, RTK-GNSS, or total-station measurements. Therefore, the reported errors for these quadruped sequences should be interpreted mainly as relative comparisons against a strong and consistent reference trajectory, rather than as strict absolute accuracy. Future work will include evaluation with externally measured ground truth to further validate the absolute accuracy of the proposed method.

Second, the proposed method includes platform-dependent hyperparameters. The clamping thresholds (ζz,ζr,ζp) and the number of temporal segments K were configured based on the motion characteristics of the Unitree Go2 platform used in this study. Although the overall framework is structurally platform-agnostic, quadruped robots with different gait patterns, body masses, walking speeds, or LiDAR mounting configurations may exhibit different levels of vertical oscillation, *roll*/*pitch* variation, and intra-scan distortion. This tendency is also reflected in the experimental results. The proposed method achieved a relatively moderate improvement on Sequence 1, which was collected using a wheeled robot on flat terrain, reducing the mean APE from 4.52 m to 1.75 m compared with KISS-ICP. In contrast, larger gains were observed on the quadruped sequences; for example, on Sequence 2, the mean APE decreased from 15.48 m to 1.34 m. These results suggest that the proposed design is particularly beneficial under legged locomotion, where the single constant-velocity assumption becomes less reliable. For other quadruped robots, bipedal robots, or different sensor configurations, platform-aware parameter adjustment may further improve performance because gait pattern, body mass, walking speed, and LiDAR mounting configuration can affect the magnitude of vertical oscillation and intra-scan distortion.

Third, the observability-aware weighting in the vertical stage depends on the availability of terrain surfaces that provide meaningful point-to-plane constraints for the vertical state. In environments dominated by vertical structures, such as narrow corridors or dense indoor scenes, or in geometrically sparse outdoor environments, the vertical observability score Ωi may remain low for most correspondences. In such cases, the advantage of the proposed decoupled vertical stage over conventional single-stage ICP may decrease, and the vertical refinement may provide limited additional benefit.

## 5. Conclusions

LiDAR-based odometry is an important front-end module for autonomous navigation of quadruped robots. However, most existing LiDAR odometry methods have been developed primarily for wheeled platforms and are therefore not well suited to the unique motion characteristics of quadruped robots, including high-frequency vibrations, abrupt postural changes, and weakened vertical observability in environments with varying elevation. These factors introduce point-cloud distortion during scan acquisition and can lead to accumulated *Z*-axis drift and unstable pose estimation. Our experiments also showed that ICP, KISS-ICP, and F-LOAM all exhibit substantially larger errors on the quadruped sequences than on the wheeled-platform sequence, highlighting the limitation of conventional LiDAR odometry under legged locomotion.

To address these issues, this paper presented an improved LiDAR odometry method for quadruped robots based on three key components. First, the proposed Piecewise-Constant Velocity deskewing divides each scan into multiple temporal segments, relaxing the limitations of a single constant-velocity assumption, while *Z*-axis and *roll–pitch* clamping suppress abnormal initial motion estimates. Second, the proposed decoupled horizontal–vertical two-stage ICP separates SE(3) optimization into planar and vertical components, preventing vertical instability from contaminating horizontal pose estimation. Third, the vertical stage introduces point-to-plane constraints together with observability-aware weighting and bounded update control to improve robustness of *Z*-axis estimation in slopes and other elevation-varying terrain.

Experiments on four sequences—S3E, DIGI, SanDan, and Playground—showed that the proposed method consistently achieved lower accumulated error and more stable trajectory estimation than ICP, KISS-ICP, and F-LOAM. Quantitatively, compared with KISS-ICP, the proposed method reduced the mean APE from 4.52 m to 1.75 m, 15.48 m to 1.34 m, 12.02 m to 2.85 m, and 11.95 m to 3.89 m on Sequences 1–4, respectively. The corresponding RMSE values were reduced from 5.38 m to 2.11 m, 20.94 m to 1.71 m, 15.32 m to 3.71 m, and 13.96 m to 4.93 m. These quantitative improvements support the conclusion that the proposed PCV deskewing and decoupled horizontal–vertical registration improve LiDAR-only odometry accuracy, with particularly clear benefits under quadruped locomotion and elevation-varying terrain.

Future work will investigate the integration of the proposed odometry front-end with back-end modules such as loop closure and pose graph optimization to improve long-range global consistency in larger environments.

## Figures and Tables

**Table 1:** Overview of the evaluated sequences.

Sequence	Dataset/ Environment	Platform	LiDAR	Slope
Seq. 1	S3E Playground	Wheel robot	Velodyne VLP-16	No
Seq. 2	DIGI	Unitree Go2	Velodyne VLP-16	Yes
Seq. 3	SanDan	Unitree Go2	Velodyne VLP-16	Yes
Seq. 4	Playground	Unitree Go2	Velodyne VLP-16	No

**Table 2:** Quantitative results (Sequence 1 from the public S3E wheeled-robot dataset; evaluated against the dataset-provided ground truth).

	Glim	Proposed	KISS-ICP	ICP	FLOAM
**Maximum (m)**	**1.088952**	4.449973	9.774980	89.017330	66.333835
**Mean (m)**	**0.576101**	1.752100	4.515873	51.924136	20.579071
**RMSE (m)**	**0.691876**	2.113185	5.376167	57.460399	29.736672
**std (m)**	**0.383146**	1.177584	2.917177	24.608567	21.465588

**Table 3:** Quantitative Results for quadruped robot sequences.

		Proposed	KISS-ICP	ICP	FLOAM
**Seq. 2**	**Maximum (m)**	**3.565001**	37.354754	72.041353	59.356439
	**Mean (m)**	**1.335711**	15.482719	29.493995	28.745720
	**RMSE (m)**	**1.712981**	20.935810	37.750446	35.238919
	**std (m)**	**1.072054**	14.092188	23.562691	20.382958
**Seq. 3**	**Maximum (m)**	**7.768837**	23.404067	57.881116	32.021459
	**Mean (m)**	**2.850228**	12.019096	36.709779	9.706683
	**RMSE (m)**	**3.707536**	15.318486	40.972650	14.937945
	**std (m)**	**2.365196**	7.771880	18.197531	11.354405
**Seq. 4**	**Maximum (m)**	**9.360022**	19.884098	59.871449	129.465068
	**Mean (m)**	**3.888720**	11.954508	38.755214	67.805222
	**RMSE (m)**	**4.932879**	13.957694	42.533199	78.637041
	**std (m)**	**3.015417**	7.204587	17.524452	39.827580

## Data Availability

The S3E dataset used in this study is publicly available from its original source. The quadruped robot datasets (DIGI, SanDan, and Go2 Playground) were collected by the authors and are available from the corresponding author upon reasonable request.
